# New insight into hybridization and unidirectional introgression between *Ammodytes japonicus* and *Ammodytes heian* (Trachiniformes, Ammodytidae)

**DOI:** 10.1371/journal.pone.0178001

**Published:** 2017-06-05

**Authors:** Jin-Koo Kim, Seung Eun Bae, Soo Jeong Lee, Moon Geun Yoon

**Affiliations:** 1 Department of Marine Biology, Pukyong National University, Busan, Korea; 2 Fisheries Resources and Environment Division, East Sea Fisheries Research Institute, National Institute of Fisheries Science, Gangnung, Korea; 3 Marine Biodiversity Institute of Korea, Seocheon-gun, Chungcheongnam-do, Korea; National Cheng Kung University, TAIWAN

## Abstract

Based on northern (NOL) and southern (SOL) mitochondrial lineages, recently, it proposed the new species *Ammodytes heian* and revived the species name *Ammodytes japonicus* to describe sand lances from the northwestern Pacific Ocean. This study used molecular methods to investigate genetic relationships between the two sand lance species in Korea and Japan. In total, 154 specimens were collected from four locations in Korea (Baengnyeongdo in the Yellow Sea, Tongyeong in the Korean Strait, and Jumunjin and Gijang in the East Sea), and 50 specimens were collected from a single location in Japan (Wakkanai in the Okhotsk Sea). Mitochondrial DNA analysis demonstrated that the individuals from Baengnyeongdo and Tongyeong all belonged to the SOL, whereas those from Gijang, Jumunjin, and Wakkanai included individuals from both the NOL and SOL (over 75% NOL). Population structure analyses were performed on the same individuals using seven microsatellite DNA markers. The population structure analysis based on 201 specimens identified two clusters (named as northern group and southern group), with the admixture proportion (q) of < 0.1 for the northern group in the Backyeongdo and Tongyeong sand lances and < 0.1 for the southern group in the Wakkanai sand lances. The high heterogeneity indicated that the former was probably *A*. *japonicus* and the latter probably *A*. *heian*. However, the admixture proportion in the Jumunjin and Gijang sand lances was 0.71–0.75 for the southern group, indicating that hybridization and unidirectional introgression from SOL to NOL occurs in southwestern margin of the East Sea. Our findings illustrate the speciation process based on different patterns of gene flow between Korean and Japanese sand lance, which is strongly influenced by both the paleo-climatic change and the contemporary local oceanic current pattern.

## Introduction

Interspecific genetic exchange and hybridization are important factors in the evolution of some species [1, 2]. Many recent studies have investigated hybridization and introgression in marine fishes [3–5]. Hybridization may lead to introgression and speciation and can also reverse or accelerate speciation [2, 6–7]. Generally, patterns of introgression vary across genomes and depend on selection (e.g., unidirectional or bidirectional selection) and the parent species [3, 8]. Turner [9] suggested that, if introgression occurs frequently, then gene flow could eventually lead to loss of distinctiveness in populations and their development into geographical variants or subspecies.

Hybridization occurs more often when the ranges of closely related species overlap [3, 5], and asymmetric introgression may take place when one species exists at low density and in sympatry with a closely related taxon [10]. Introgression by unidirectional gene flow can lead to a population losing its genetic identity, and bidirectional introgression can reverse speciation [11].

Cryptic species, which are morphologically identical but genetically distinct, may be generated allopatrically or sympatrically. However, genetic identification of cryptic species can be considerably more difficult when they have recently diverged by introgression [12, 13]. The northwestern Pacific sand lance is one species that could have resulted from introgressive hybridization or directional introgression because the possibility of hybridization between species of the taxonomically revised genus *Ammodytes* remains.

The taxonomic status of the northwestern Pacific sand lance was first questioned by Turanov and Kartavtsev [14], who suggested it was necessary to revise the scientific name *Ammodytes personatus* when referring to sand lances inhabiting the temperate zone of the Yellow Sea, the southern East Sea (Sea of Japan), and the waters south of Japan. Previous morphological and genetic studies on the Korean *A*. *personatus* suggested there were two lineages: (1) a northern lineage (NOL) and (2) a southern lineage (SOL) [15–17]. The number of vertebrae was a good taxonomic characteristic for distinguishing the two lineages: although the numbers partially overlap [16], 64–67 (modes = 65 and 66) in the NOL from the East Sea, and 62–65 (modes = 62, 63, and 64) in the SOL from the Yellow Sea and the Korean Strait. However, the NOL and SOL could not be regarded as separate species because of the presence of an admixture zone [18], spawning aggregation [19], and gene flow between the two lineages [20].

Nonetheless, the two lineages can be distinguished by their mitochondrial DNA (mtDNA) cytochrome oxidase I (COI) sequences, and Orr et al. [21] described them as separate species. That study proposed that the NOL from Fukushima and Wakkanai in Japan was a new species, *Ammodytes heian*, and Orr et al. [21] revived the species name *Ammodytes japonicus* to describe the SOL from the same area and southern Japan. Orr et al. [21] described the different morphological characteristics of the two species: the number of pored lateral-line scales (142–185 in *A*. *heian* vs. 132–166 in *A*. *japonicus*), the number of dorsal plicae (159–198 in *A*. *heian* vs. 144–182 in *A*. *japonicus*), body height (40–57% in *A*. *heian* vs. 32–53% in *A*. *japonicus*), upper jaw length (29–32% in *A*. *heian* vs. 30–39% in *A*. *japonicus*), and eye diameter (11–15% in *A*. *heian* vs. 12–20% in *A*. *japonicus*). However, these ranges overlap, making it difficult to distinguish between the two species using morphological features alone.

Many studies have used highly polymorphic microsatellite DNA markers to identify both individuals and populations [19, 20, 22]. Microsatellites are generally more variable than mtDNA and allozymes, therefore more useful for population genetic studies that include fingerprinting and genetic mapping [20, 23, 24]. In marine organisms, they can be used to analyze genetic diversity between wild and farmed organisms, verify parentage, and quantify population genetic variation in related and hybrid species [20, 25–27].

The aim of this study was to make comprehensive genetic comparisons that would reveal relationships among Korean and Japanese sand lances and to understand their microevolutionary history, which might include hybridization or unidirectional introgression.

## Materials and method

### Sample collection

**Fig 1:**
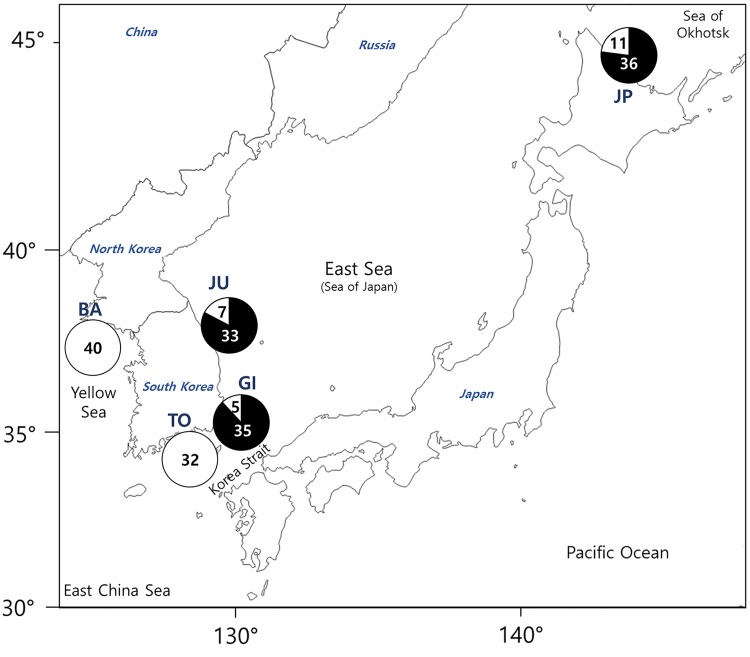
Map showing the occurrence ratio of each lineage in Korean and Japanese sand lance. Four locations in Korea: BA (Baengnyeongdo, n = 40), TO (Tongyeong, n = 32), GI (Gijang, n = 40), JU (Jumunjin, n = 40), and one location in Japan: JP (Wakkanai, n = 47). Open and solid circles indicate southern lineage (SOL) and northern lineage (NOL), respectively.

This research did not involve endangered or protected species, and any locations, which performed in this research, are not required for specific permission. A total of 204 adult individuals were collected at four locations around Korean peninsula and one location in Japan during 2010–2015 (Fig 1). The sampling areas and dates are shown in Table 1. Muscle tissues were fixed and preserved in 95% ethanol. All Korean samples were deposited at the Ichthyology Laboratory in Pukyong National University (PKU), Korea.

**Table 1:** Collection information of sand lance specimens used in this study.

Sampling area	Sampling date	Number of individuals		Voucher number
		Southern lineage	Northern lineage	
Baengnyeongdo, Korea	21 May, 2015	40	0	PKU11866-11915
Tongyeong, Korea	10 Jun., 2010 24 Aug., 2010	34	0	PKU3626-3655, 4076–4079
Gijang, Korea	31 Mar., 2011	5	35	PKU11717-11726, PKU11730-11739 PKU11741-11760
Jumunjin, Korea	24 Nov., 2013	7	33	PKU11767-11791, PKU11793-11795 PKU11797-11804, PKU11808-11811
Wakkanai, Japan	Jun., 2015	11	36	PKU13232-13281

### DNA extraction, PCR and sequencing

Genomic DNA was extracted from muscle tissue or pectoral fin clips using the AccuPrep Genomic DNA extraction Kit (Bioneer, Daejeon, Korea). Partial fragments of mitochondrial DNA cytochrome c oxidase subunit I (mtDNA COI) were amplified using VF2 and FishR2 (5′-TCW ACC AAC CAC AAA GAY ATY GGG AC-3′) primers [28]. We carried out the PCR under the following conditions: initial denaturation for 5 min at 95°C, 35 cycles (denaturation for 1 min at 95°C, annealing for 1 min at 52°C, extension for 1 min at 72°C) and final extension for 5 min at 72°C. The amplified PCR product was electrophoresed in 1% agarose gel and visualized by an UV illuminator. PCR products were purified the using AccuPrep^®^ PCR purification kit (Bioneer, Korea) and directly sequenced using the ABI BigDye terminator cycle Sequencing Ready Reaction Kit v.3.1 (Applied Biosystems Inc., USA). The mtDNA COI sequences were aligned using ClustalW [29] in BioEdit v.7 [30]. The mtDNA COI Sequences were registered to the DDBJ / EMBL / GenBank nucleotide sequence databases (accession numbers KY773707–KY773908).

### Microsatellite amplification

We designed seven microsatellite primers (Ape341, Ape327, Ape104, Ape308, Ape315, Ape349, Ape302) according to the methods of Ren et al. [31]. The 5’-end of the forward primer for each primers was labeled with a fluorescent dye (6-FAM, HEX; Applied Biosystems, USA). PCR amplifications were carried out in 12.5μL reaction volumes contacting 50-100ng template DNA, 1.25 μL 10X Ex taq buffer, 2.5mM dNTP Mixture, 10μm of each primer, and 0.1μL Taq polymerase (TaKaRa, Shiga, Japan), and PCR conditions was performed according to Ren et al. [20]. Successful amplification was assessed by electrophoresis using 1% agarose gel to separate DNA fragments. PCR products were diluted with deionized water, combined with a mixture of Hi-Di^™^ Formamide (Applied Biosystems) and internal size standard (GENESCAN 400HD ROX; Applied Biosystems) and denatured for 2 minutes at 95°C. Samples were run on an ABI 3730xL DNA sequencer and fluorescent peak data were sized and scores using Genemapper ver. 4.0 (Applied Biosystems, USA).

### Data analysis

For mtDNA COI sequences, the number of haplotypes, haplotype frequency and pairwise FST were calculated using Arlequin v.3.5.1.2 [32]. A minimum spanning network was constructed from the matrix of pairwise distances calculated among all haplotypes using Arlequin v.3.5.1.2, and TCS v.1.21 program. The phylogenetic tree was constructed with the neighbor-joining method and 1,000 bootstrap replications in MEGA 7. The sequences of *A*. *japonicus* (KJ137283 of Orr et al. [21]), *A*. *heian* (KJ137282 of Orr et al. [21]), *A*. *personatus* (KJ137281 of Orr et al. [21]), and *A*. *hexapterus* (KJ137280 of Orr et al. [21]) from the National Center for Biological Information database were used to confirm the taxonomic status of the Korean and Japanese sand lances.

For microsatellite analysis, in order to analyze genetic diversity for each locus, we calculated the number of allele (Na), observed heterozygosity (Ho), expected heterozygosity (He) and polymorphic information content (PIC) of the seven microsatellite DNA markers using Cervus v. 3.0.6 [33] and calculated allelic richness (Ar) adjusted by population size and inbreeding coefficient (FIS) using FSTAT v. 2.9.3.2 (https://www2.unil.ch/popgen/softwares/fstat.htm). Hardy-Weinberg equilibrium within a population was verified using GENEPOP v. 4.3 [34, 35].

Genetic boundaries between populations were inferred using BARRIER software version 2.2 [36]. For genetic population structure, FST values were calculated using Arlequin v.3.5.1.2. To investigate the pattern of population structure, the software STRUCTURE v.2.3.4 [37] was used to identify clusters of genetically similar populations based on Bayesian approach. To infer most likely number of populations values of K (K = 1–7) were evaluated using 10 independent runs for each K value. Each replicate run consisted of 1,000,000 Markov Chain Monte Carlo (MCMC) repetitions; a burn-in 100,000 iterations to calculate inferred average values and standard deviations. To determine the optimum K value, ΔK was calculated using STRUCTURE HARVESTER [38]. Discriminant analysis of principal components (DAPC) also provided posterior probabilities of population assignment for each individuals [39]. To estimate the contemporary migration rates were estimated using Migrate-n version 3.6.11 [40]. We set datatype to Microsatellite, migration prior (FST), constant mutation rate with a stepwise mutation, migration rate parameter (Theta and M to a maximum of 10), and a Bayesian analysis.

## Results

### MtDNA

**Fig 2:**
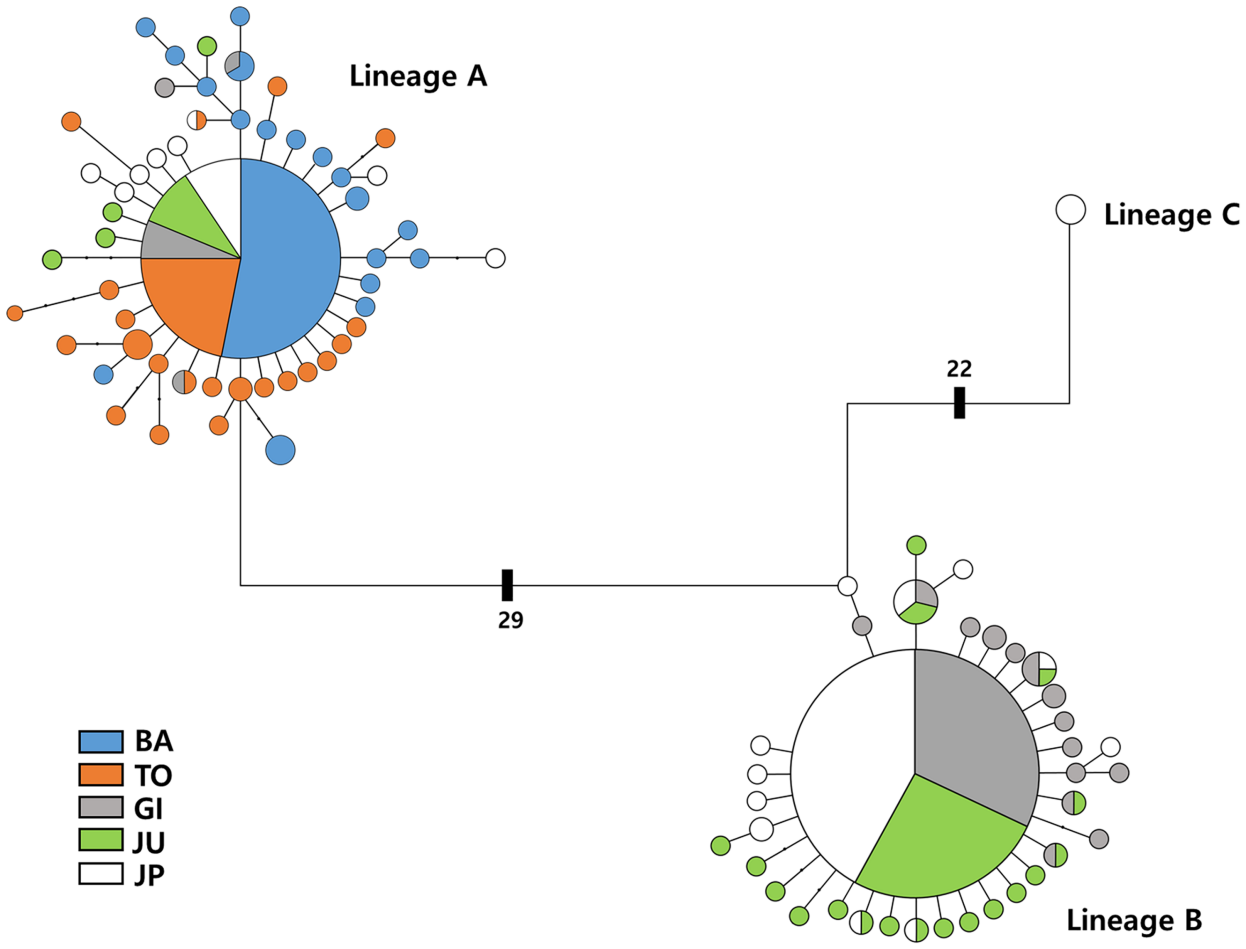
Minimum-spanning network of mtDNA COI haplotypes in Korean (4 locations) and Japanese (1 location) sand lance. The size of each circle is proportional to the haplotype frequency, and the branch lengths are proportional to the mutational steps. Lineages A, B and C correspond to *Ammodytes japonicus*, *A*. *heian* and *A*. *hexapterus* of Orr et al. (2015).

MtDNA sequences were used to categorize the 202 individual specimens collected from the five locations (Baengnyeongdo, Tongyeong, Gijang, and Jumunjin in Korea and Wakkanai in Japan). At Baengnyeongdo in the Yellow Sea and Tongyeong in the Korean Strait, only SOL specimens were collected, whereas specimens from both the SOL and NOL were collected at Gijang and Jumunjin in the East Sea and Wakkanai in the Okhotsk Sea, with NOL being dominant (> 75% of the total) (Fig 1). In total, 89 haplotypes were identified, forming three distinct clades were detected in the minimum spanning network analysis. The first clade (lineage A) consisted of individuals from all locations. The second clade (lineage B) consisted of individuals from three locations (Gijang, Jumunjin, and Wakkanai). The third clade (lineage C) consisted of only one haplotype, and all of these specimens were from Wakkanai (Fig 2). Compared to sequences of our *Ammodytes* species with those of Orr et al. [21], the phylogenetic tree showed that lineages A, B, and C corresponded to *Ammodytes japonicus*, *A*. *heian*, and *A*. *hexapterus*, respectively (S1 Fig).

### Microsatellite DNA

Seven microsatellite markers were used for genetic analysis of the 201 specimens. This analysis determined the number of alleles (Na), observed (Ho) and expected (He) heterozygosity, polymorphic information content (PIC), probability value estimates for deviations from Hardy—Weinberg equilibrium, and inbreeding coefficients (FIS) (Tables 2 and 3). Average number of alleles (Na) ranged from 22.8 (Ape 104) to 34.4 (Ape 349). Allele frequencies for each locus showed that Japanese sand lance has both the highest in Ape 304 (22.5%) and the lowest frequency in Ape 341 (8.09%) (S2 Fig). Heterozygosity was high with Ho values ranging from 0.564 (Ape 308) to 0.912 (Ape 104), and with He values varying from 0.885 (Ape 308) to 0.972 (Ape 349). PIC was high in most locations, but the Japan was the lowest values (0.870). Overall genetic variability was high, with allelic richness (Ar) per samples averages ranging from 20.4 (Japan) to 26.2 (Gijang). At population level, both Ho and He values was shown in the lowest Japan (Ho = 0.728, He = 0.885), but the highest in Jumunjin (Ho = 0.770, He = 0.961). In HWE test, high level of HW disequilibrium was observed in most of seven loci. Three of the seven loci were found to be out of HWE in Japanese population unlike Korean population having the departures from HWE in one locus, and this might be due to relatively small number of populations in Japan (Tables 2 and 3).

**Table 2:** Genetic variation at seven microsatellite loci in Korean and Japanese sand lances.

Location		Locus							Mean
		Ape341	Ape327	Ape349	Ape302	Ape104	Ape308	Ape315	
Baengnyeongdo [BA]	N	39	37	39	38	39	38	39	
	Na	25	24	33	26	24	41	35	29.71
	Ho	0.667	0.514	0.795	0.711	0.974	0.632	0.821	0.731
	He	0.946	0.953	0.971	0.944	0.947	0.976	0.971	0.958
	PIC	0.930	0.937	0.957	0.927	0.931	0.962	0.957	0.943
	P	0.0000	0.0000	0.0000	0.0000	0.3655	0.0000	0.0000	
	FIS	0.298	0.465	0.183	0.249	-0.03	0.356	0.156	0.240
	Ar	21.4	21.6	28.3	22.3	21.3	33.4	29.1	25.4
Gijang [GI]	N	38	40	40	40	40	40	40	
	Na	31	28	36	31	24	37	28	30.71
	Ho	0.5	0.85	0.85	0.725	0.95	0.5	0.725	0.729
	He	0.969	0.951	0.974	0.962	0.953	0.972	0.959	0.963
	PIC	0.955	0.936	0.961	0.947	0.938	0.958	0.945	0.949
	P	0.0000	0.0558	0.0009	0.0000	0.7774	0.0000	0.0005	
	FIS	0.487	0.107	0.129	0.249	0.003	0.489	0.247	0.244
	Ar	27.2	23.4	30.3	26.0	21.5	30.7	24.0	26.2
Jumunjin [JU]	N	35	40	40	40	40	39	39	
	Na	26	26	32	29	23	39	34	29.86
	Ho	0.429	0.875	0.925	0.725	0.975	0.615	0.846	0.770
	He	0.952	0.955	0.971	0.96	0.953	0.975	0.968	0.962
	PIC	0.935	0.94	0.957	0.946	0.938	0.961	0.954	0.947
	P	0.0000	0.1464	0.4314	0.0000	0.6118	0.0000	0.0117	
	FIS	0.553	0.085	0.048	0.247	-0.024	0.372	0.127	0.201
	Ar	23.8	23.2	27.7	24.7	20.6	32.1	28.8	25.8

Total number of alleles (N), average number of alleles (Na), observed (H_O_) and expected (H_E_) heterozygosities, polymorphic information content (PIC), probability value estimates regarding deviation from Hardy—Weinberg equilibrium (*P*), inbreeding coefficient (F_IS_) and allelic richness.

**Table 3:** Genetic variation at seven microsatellite loci in Korean and Japanese sand lances (Table 2 continued).

Location		Locus							Mean
		Ape341	Ape327	Ape349	Ape302	Ape104	Ape308	Ape315	
Tongyeong [TO]	N	32	28	32	27	33	32	31	
	Na	22	22	32	20	24	31	24	25.00
	Ho	0.813	0.571	0.719	0.852	0.939	0.5	0.71	0.729
	He	0.933	0.955	0.971	0.957	0.956	0.967	0.936	0.954
	PIC	0.913	0.934	0.954	0.936	0.939	0.95	0.917	0.935
	P	0.0091	0.0000	0.0000	0.0052	0.4157	0.0000	0.0024	
	FIS	0.131	0.406	0.263	0.112	0.018	0.487	0.245	0.237
	Ar	20.7	21.8	29.6	20.0	22.3	28.5	22.6	23.6
Japan [JP]	N	47	46	46	47	43	47	40	
	Na	14	22	39	34	19	19	26	24.71
	Ho	0.851	0.783	0.913	0.702	0.721	0.574	0.55	0.728
	He	0.869	0.951	0.974	0.965	0.938	0.535	0.961	0.885
	PIC	0.845	0.937	0.962	0.953	0.923	0.522	0.947	0.870
	P	0.6206	0.0000	0.0246	0.0000	0.0016	0.9381	0.0000	
	FIS	0.021	0.178	0.063	0.274	0.234	-0.075	0.431	0.161
	Ar	11.7	20.0	30.3	26.7	17.1	13.6	23.4	20.4

Total number of alleles (N), average number of alleles (Na), observed (H_O_) and expected (H_E_) heterozygosities, polymorphic information content (PIC), probability value estimates regarding deviation from Hardy—Weinberg equilibrium (*P*), inbreeding coefficient (F_IS_) and allelic richness.

**Fig 3:**
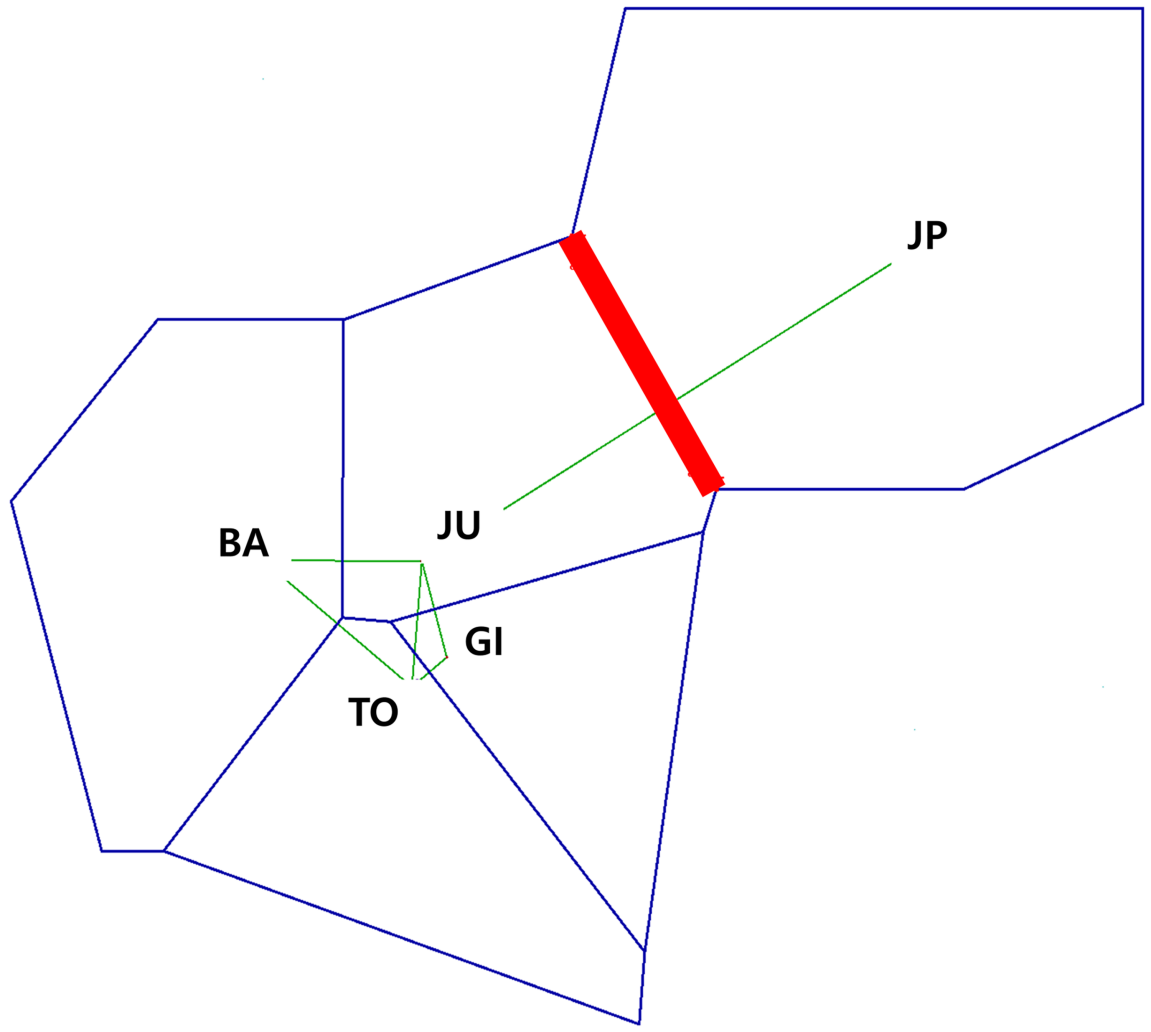
Result of the BARRIER analysis based on FST matrices from seven microsatellite. The red line shows detected a barrier. Each population is indicated by abbreviation; Baengnyeongdo (BA), Tongyeong (TO), Gijang (GI), Jumunjin (JU) and Japan (JP), respectively.

**Fig 4:**
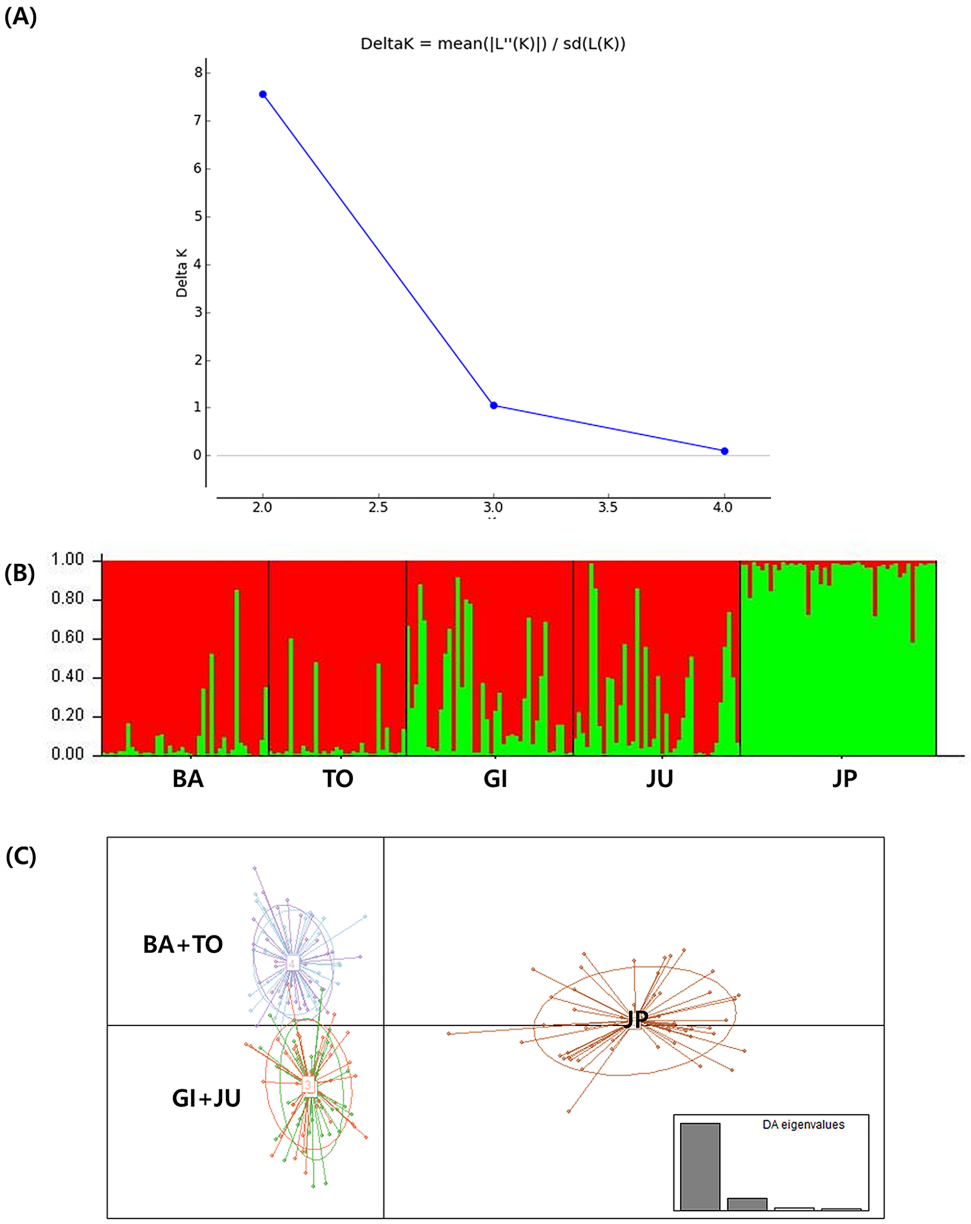
Population structure of sand lances based on seven microsatellite loci. (A) Estimation of population clustering level from seven microsatellite genotypes following Evanno et al. (2005) criteria obtained from STRUCTURE HARVESTER. (B) Population structure inferred by Bayesian clustering method using the STRUCTURE program. The different colors represent two different clusters (K = 2). Vertical lines separate each lineage of 5 locations. (C) Scatterplots of the discriminant analysis of principal components of the microsatellite data for 5 locations. Individual genotypes appear as dots. Each population is indicated by abbreviation; Baengnyeongdo (BA), Tongyeong (TO), Gijang (GI), Jumunjin (JU) and Japan (JP), respectively.

Values of pairwise FST were significant for comparisons between the Baengnyeongdo individuals and those in Gijang, Jumunjin, and Wakkanai, similarly comparisons between Wakkanai and all the Korean locations were significant (Table 4; S1 Table). BARRIER analysis demonstrated a genetic barrier between the Korean and Japanese sand lance, supported by FST values (FST > 0.05, Fig 3). According to the STRUCTURE analysis, the ΔK value was greatest at K = 2 (Fig 4A). These results indicate that the sand lances inhabiting Korea and Japan represent two distinct genetic units. The structure plots had two clusters with ratios that varied depending on location. The individuals from Wakkanai occurred primarily in cluster 2 (> 90%), whereas those from the four Korean locations were found predominantly in cluster 1. Specimens from Baengnyeongdo and Tongyeong consisted mostly of individuals from cluster 1 (> 90%), but only 70.9–75.0% of specimens from Gijang and Jumunjin were from cluster 1 (Fig 4B; Table 5). The first axis of the DAPC separated the Korean and Japanese sand lances into two spatially distinct populations (Fig 4C). This result was consistent with the STRUCTURE analysis. In addition, the UPGMA tree showed that sand lances were divided into two distinct groups (Korean group and Japanese group), and Korean group was separated into two subgroups (Baengnyeongdo + Tongyeong subgroup and Jumunjin + Gijang subgroup) (S3 Fig). Therefore, the sand lances were separated into Korean (Baengnyeongdo, Tongyeong, Gijang, and Jumunjin) and Japanese (Wakkanai) populations. As displayed in Table 6, the highest levels of gene flow between locations was observed from Tongyeong to Gijang (Nm = 71.00), and the lowest from Jumunjin to Tongyeong (Nm = 18.33). In addition, the level of gene flow between Jumunjin and Wakkanai had low values (Nm = 19.00). This indicated that asymmetrical gene flow between two group, and a minimal amount of gene flow between the Korean and Japanese sand lances, consistent with the BARRIER analysis.

**Table 4:** Pairwise FST values between the five locations (below diagonal) and pairwise *P* values (above diagonal) in seven microsatellite loci.

Location	Baengnyeongdo	Tongyeong	Gijang	Jumunjin	Japan
Baengnyeongdo		-	+	+	+
Tongyeong	0.0041		-	-	+
Gijang	0.0093	0.0059		-	+
Jumunjin	0.0086	0.0022	0.0016		+
Japan	0.0619	0.0618	0.0564	0.0561	

+, significant.

−, not significant in FST.

Significance was tested at the *P* < 0.01.

**Table 5:** The number of sand lance individuals in each location and proportion of individuals in two clusters inferred by STRUCTURE analysis based on seven microsatellite loci.

Location	Inferred clusters		Number of individuals
	Cluster 1	Cluster 2	
Baengnyeongdo	0.915	0.085	40
Tongyeong	0.926	0.074	33
Gijang	0.709	0.291	40
Jumunjin	0.750	0.250	40
Japan	0.052	0.948	47

**Table 6:** Relative gene flow rate among five locations of sand lance based on seven microsatellite loci.

From location	To location	Nm
Gijang	Baengnyeongdo	33.67 (9.33–54.67)
Jumunjin	Baengnyeongdo	46.33 (24.00–66.00)
Tongyeong	Baengnyeongdo	43.00 (17.33–64.67)
Japan	Baengnyeongdo	51.00 (16.00–75.33)
Baengnyeongdo	Gijang	23.00 (1.33–4.672)
Jumunjin	Gijang	23.67 (0.67–44.67)
Tongyeong	Gijang	71.00 (42.67–100.00)
Japan	Gijang	39.00 (12.67–65.33)
Baengnyeongdo	Jumunjin	51.67 (27.33–74.67)
Gijang	Jumunjin	21.67 (0.00–45.33)
Tongyeong	Jumunjin	21.67 (0.00–42.00)
Japan	Jumunjin	45.67 (18.00–72.00)
Baengnyeongdo	Tongyeong	38.33 (14.00–61.33)
Gijang	Tongyeong	21.00 (1.33–38.00)
Jumunjin	Tongyeong	18.33 (0.00–34.00)
Japan	Tongyeong	27.00 (0.00–59.33)
Baengnyeongdo	Japan	25.00 (5.33–44.00)
Gijang	Japan	32.33 (4.00–61.33)
Jumunjin	Japan	19.00 (0.00–34.67)
Tongyeong	Japan	27.00 (3.33–48.00)

Values in brackets represent the 95% confidence values.

## Discussion

### Historic divergence vs. contemporary gene flow

A minimum spanning network analysis based on mtDNA COI sequences identified three distinct lineages (A, B, and C) among the Korean and Japanese sand lances. The genetic distances between these lineages were 7.2–8.5% (A—B), 7.7–8.5% (A—C), and 5.4–6.2% (B—C), respectively. Compared with the study findings reported by Orr et al. [21], lineages A, B, and C correspond to *Ammodytes japonicus*, *A*. *heian*, and *A*. *hexapterus*, respectively. The mtDNA results demonstrate that *A*. *japonicus* is distributed throughout Japan, China, and Korea, but the distribution of *A*. *heian* is limited to Korea and Japan, and that of *A*. *hexapterus* is restricted to Japan. The distribution of *A*. *japonicus* and *A*. *heian* partially overlap in the southwestern margin of the East Sea and in Wakkanai, suggesting the possibility of hybridization between these two species. A more powerful analytical procedure using microsatellite DNA markers will be required to define these relationships. The microsatellite markers demonstrated that all populations had a high degree of genetic variability (He = 0.885–0.963), and the pairwise FST values were significantly different among populations (FST = 0.028, P < 0.01). The STRUCTURE analysis demonstrated that the sand lances were clustered into two groups, but the structure plot indicated that all individuals included mixed ratios from each cluster. The Wakkannai sand lance consisted of more than 90% cluster 2 (northern group), whereas the Baengnyeongdo and Tongyeong sand lances each consisted of more than 90% cluster 1 (southern group), suggesting there may be very little gene flow between the northern group (Wakkannai, Japan) and the southern group (Baengnyeongdo and Tongyeong, Korea). In addition, DAPC plots showed genetically two groups corresponding to Japanese and Korean sand lance and this is consistent with that of the BARRIER analysis. According to Palero et al. [41], the DAPC analysis in European spiny lobster (*Palinurus Elephas*) showed a clear split into the Atlantic and Mediterranean populations, suggesting the barriers between the Atlantic and Mediterranean transition. Therefore, our results indicated the presence of strong boundary between the Korean and Wakkanai sand lances. Additionally, Ren et al. [20] suggested that the northern group of sand lances is mostly distinct from the southern group except those in Kashima and Mutsu Bay and that difference in the allele frequencies between the two groups might indicate very limited gene flow between the two. Using cytochrome b and microsatellite analyses, Muths et al. [42] demonstrated significant genetic differentiation between *Ephinephelus merra* populations living in the western Indian Ocean, which suggested the presence of a cryptic lineage. In this study, both the mtDNA and microsatellite analyses indicated significant genetic variation among the sand lances.

In agreement with our results, previous studies using mtDNA COI (genetic distance, d = 8.1%) and control region analysis (d = 5.8%) have demonstrated that the Korean sand lance is highly differentiated at different locations [15, 19]. Indeed, movement of the two distinct mtDNA COI lineages was restricted by geological barriers. Various studies have suggested that the genetic structure of marine organisms living in the northwestern Pacific Ocean was affected by Pleistocene glaciation [15, 43, 44]. The lowered sea level during periods of glaciation probably isolated the East Sea (Sea of Japan) from the rest of the northwestern Pacific Ocean, perhaps resulting in historic divergence and a high level of local adaptation of the two lineages. Also, Kim et al. [19] suggested that both SOL and NOL larvae are limited distribution due to a physical barrier formed by the Nakdong River as well as because of the intersection between two different oceanic currents (the Eastern Korea Warm Current and North Korean Cold Current). However, spawning areas and timing for the two sand lance lineages overlap considerably in the southwestern margin of the East Sea, where NOL and SOL larvae ratios are similar, and this indicated possibility of gene flow between the two lineages. In this study, most of loci showed high level of Hardy Weinberg disequilibrium, and especially, two loci (Ape302 and Ape 315) showed significant deviations from HWE in all populations. Ren et al. [31] found the deviations from HWE in some loci, and suggested that these could be affected by null alleles. In addition, Ren et al. [20] found the presence of null allele in locus or the dropout of large allele in the 12 deviation from HWE populations. In this study, the frequency of null allele ranged from 0.00 to 0.26, indicating the deviation from HWE could be associated with presence of null alleles. However, Ren et al. [31] insisted that these ms markers are useful for population structure study of sand lance despite presence of null alleles. Furthermore, recent study revealed that null alleles could not change the overall result of population structure using ms loci [45]. The HW disequilibrium due to a deficiency of heterozygotes could be resulted by various factors such as inbreeding, selection, genetic drift or Wahlund effect [46–48]. The extent of HWE depends on migration pattern, selection, and isolation between two groups [49]. The population structure of Killifish (*Fundulus heteroclitus*) using ms markers showed two distinct groups, which were consistent with previous study (mtDNA and allozyme loci), and the distribution and population structure between two groups were associated with differences of allele frequencies by fixation of clinal alleles [50]. In case of herring in the Baltic Sea, clinal patterns of genetic differentiation between Baltic and Atlantic region occurred, and this pattern was related with higher or lower frequencies in certain alleles. Similarly, in this study, Japanese (Wakkanai) and Korean (Baengnyeongdo and Tongyeong) sand lance have well-defined groups in both mtDNA and ms loci, and this could be interpreted as two genetically heterogenous groups by allele fixation. According to Mallet [51], heterozygote deficiencies and linkage disequilibrium could be indicate that multiple distinct species have been pooled within a population. In case of brown trout *Salmo trutta*, putative hybrid populations have higher HW disequilibrium than pure ones, and these disequilibrium could be evidence of highly subdivided genetic structure [52]. In addition, Bossu et al. [53] suggested that if there is a random mixing between genetically distinct two darter species (*Etheostoma bison* and *E*. *caeruleum*), linkage disequilibrium is expected to be high, and the more frequencies of hybrids have tendency to the more significant deviation from HWE. In this study, although two groups of sand lance have little gene flow, there are evidence of gene flow between two groups in the southwestern margin of the East Sea, indicating that genetically effective migration may occur. Especially, Gijang and Jumunjin sand lances have higher frequencies of mixing through gene exchange by migration, and this could affect their allele frequency and deviations from HWE. Therefore, this departures from HWE could be related to the several factors including the migration. In addition, the microsatellite analyses in this study that indicated the presence of gene flow also suggest that sand lances from the southwestern margin of the East Sea (Jumunjin and Gijang) have a less well-defined genetic structure. This may be explained by a variety of evolutionary mechanisms, including incomplete sorting of ancestral polymorphisms, and sex-biased hybridization [54]. Consistent with our study, Han et al. [18] reported that secondary contact may occur between the two sand lance lineages in the waters surrounding Hokkaido and the Iwate Prefecture of Japan, where the Oyashio Cold Current and the Kuroshio Warm Current meet and the fish have poorly defined genetic structure due to the flow of currents. Ren et al. [20] also suggested that the oceanic currents may provide the opportunity for connectivity of among populations. Therefore, oceanic current fluctuations can have a significant influence on genetic exchanges between populations, and oceanographic processes may affect both adult migration and larval dispersal. Topographical factors are one possible explanation. The Korean Strait that separates Korea from Japan is very wide (175 km), whereas the Tsugaru Strait between Honshu and Hokkaido is relatively narrow (53 km), and gene flow between the two lineages across the Korean Strait was probably more common during the glaciation period. Therefore, the current population structure may be explained by both historical recolonization and current gene flow resulting from larval dispersal by oceanic currents.

### Unidirectional introgression

Our results demonstrated that 71–75% of the Gijang and Jumunjin sand lances (mainly belong to NOL in mtDNA results) were from cluster 1 (southern group). The migration rate inferred from microsatellite markers was highest between Tongyeong (all comprise SOL) and Gijang (mostly comprise NOL), accordingly, unidirectional introgression occurs predominantly from the SOL (Yellow Sea, Korean Strait) to the NOL (East Sea). Han et al. [18] discovered admixture zones between the two lineages in the waters surrounding Hokkaido and the northeastern coast of Honshu in Japan, suggesting that secondary contact did occur. The admixture proportions (q) in Kashima (Iwate Prefecture) and Mutsu Bay were 0.226 and 0.105, respectively, suggesting that reproductive isolation between the two groups was not complete [20]. In Bayesian admixture plot, most of the Icelandic eels showed on-going hybridization between American and European eels unlike having only a typical American haplotype in mtDNA, and this might be due to asymmetric introgression followed by subsequent backcrossing [55]. Similarly, most individuals of *Merluccius capensis* and *M*. *paradoxus* from North Benguela have mixed genotypes equivalent to 0.36 and 0.2, respectively. In particular, all *M*. *paradoxus* individuals had *M*. *capensis* COI sequences, and this resulted from recurrent backcrosses between *M*. *capensis* × *M*. *paradoxus* hybrid females and *M*. *paradoxus* [56]. Therefore, backcrossing between descendants of *A*. *japonicus* × *A*. *heian* hybrid females and *A*. *japonicus* males could have occurred in Gijang and Jumunjin.

Hybridization occurs frequently when spawning areas or periods overlap [3, 5, 57, 58]. Bae et al. [5] describe how sea bass (*Lateolabrax japonicus*) and spotted sea bass (*Lateolabrax maculatus*) have overlapping distributions in the Korean Strait and how some intermediate individuals (*Lateolabrax* sp.) of mixed genetic composition were formed by unidirectional introgression. Additionally, two North American Atlantic hake species, *Merluccius bilinears* and *M*. *albidus*, demonstrated a high level of introgressive hybridization where their ranges overlapped, suggesting that long-term hybridization and backcrossing had occurred [56]. Kim et al. [19] suggested that the spawning areas of the two lineages of Korean sand lance overlap considerably in the southern East Sea, and the unidirectional hybridization between these two groups is feasible.

Hybridization has great potential for rapidly introducing variability into recipient populations if the barriers to recombination can be overcome [55]. For example, *Serranus cabrilla* individuals in Cabo de Gata (CG) and Cabo de Palos (CP) from the Atlantic group were comparable to 21.7% of fish with an admixed genotype from a Mediterranean population. This may have occurred when migrants were carried south by oceanic currents. Therefore, genetic population structures may be significantly influenced by present current regimes that traverse oceanographic barriers [59]. Strong directional gene flow may occur where there is dispersal in one direction due to selective barriers. Kim et al. [19] suggested that SOL larvae may be readily dispersed from the Yellow Sea to the Korean Strait by the Western Korea Cold Waters, while NOL larvae may not be transported from the East Sea southwest to the Korean Strait against the Eastern Korea Warm Current prevailing in the surface layer; indeed, this could facilitate unidirectional introgression between the two genotypes despite the weak biogeographical barrier between the Yellow Sea / Korean Strait (Baengnyeongdo and Tongyeong) and East Sea (Jumunjin and Gijang). Therefore, the present genetic structure of marine organism populations may be heavily influenced by local oceanic currents. The seasonal and directional changes of a variety of fronts and currents may be extremely important in determining gene flow patterns and population structure [59, 60].

Hybridization can also occur between species when one species is rare and individuals are forced to find mates from a closely related species [2, 61]. Kim et al. [19] reported that the number of sand lance larvae decreased significantly in the southern East Sea. Therefore, it is possible that interspecific hybridization occurs in preference to assortative mating when there are few individuals from a particular group. Interspecific hybridization driven by low numbers of individuals might be common throughout the hybrid zone in the southwestern margin of the East Sea (Gijang and Jumunjin).

Another cause of asymmetric gene flow is sex-biased dispersal or philopatry. Different genetic patterns in maternally inherited markers (e.g., mtDNA) and bi-parentally inherited markers (e.g., microsatellites) may arise because of sexual differences in spawning behavior or simply by genetic drift and population bottlenecks [62]. If female-biased dispersal occurs, mtDNA will be homogeneous, but nuclear DNA (nDNA) will exhibit differentiation. In contrast, if male-biased dispersal occurs, mtDNA will differentiate, but nDNA will not [63]. Therefore, further studies will be required to determine whether sex-biased dispersal has a role to play in these relationships.

### Taxonomic implications

Orr et al. [21] identified two mitochondrial lineages (the NOL and SOL) in sand lances from Fukushima (Iwate Prefecture) and Wakkanai (Hokkaido). They proposed that the NOL corresponded to the new species *Ammodytes heian* and revived the species name *Ammodytes japonicus* to describe the SOL. Orr et al. [21] reported that these two species could be morphologically distinguished by the number of pored lateral-line scales, the number of dermal plicae, body height, upper jaw, and eye diameter. However, these characteristics were overlapped. Baltic herring differ regionally in their weight, length, number of vertebrae, and number of pectoral fin rays, and these variations may reflect phenotypic plasticity due to genetic differentiation in addition to differences in environmental conditions during their development [64]. Therefore, morphological variations between southern and northern groups of sand lance may certainly be influenced by environmental factors (e.g., temperature) in addition to genetic ones.

The two lineages were regarded as separate species [21], probably because the genotypes of Japanese sand lances were mostly consistent with haplotypes from the all same areas except two areas (Kashima, Mutsu bay) [20]. However, it is difficult to conclude that the two lineages of Korean sand lances are distinct species because gene flow occurs between the two in Gijang and Jumunjin. Kai et al. [65] performed amplified fragment length polymorphism marker analysis on three lineages (named as SOJ1, SOJ2 and SOJ3) of *Careproctus rastrinus* living in the northwestern Pacific Ocean and demonstrated that SOJ1 (group 1 in Sea of Japan) and SOJ2 (group 2 in Sea of Japan) were not separated by principal component analysis, suggesting that gene flow may have occurred by secondary contact. The study concluded that the two lineages (SOJ1 and SOJ2) were not cryptic species because, although their mtDNA was well differentiated, they could not be separated by nDNA analysis. Therefore, it is unlikely that the two sand lance lineages in the southwestern margin of the East Sea are cryptic species because they could not be differentiated by microsatellite analysis (Table 5, Fig 4). Instead, by being exposed to secondary contact before reaching complete reproductive isolation, the two sand lance lineages in the southwestern margin of the East Sea may be evolving toward genetic homogeneity.

Speciation generally occurs as a gradual process, by reproductive isolation and genetic differentiation between populations [66]. Hybridization and introgression occur when reproductive isolation is incomplete. Although the NOL and SOL have different morphotypes [16], ontogenetic characteristics [17], and hatching types [67], it cannot be concluded that these two lineages in the southwestern margin of the East Sea have been completely separated by reproductive isolation. Based on this study and a comprehensive review of many previous ones, we believe the sand lance in the Yellow Sea and Korean Strait is *A*. *japonicus*, whereas the sand lance in the southwestern margin of the East Sea may be derived from the backcrosses between descendant of *A*. *japonicus* × *A*. *heian* hybrid females and *A*. *japonicus* males because of the significantly higher proportion of admixture (0.71–0.75). The present study serves as a follow-up study which confirms the hypotheses of Han et al. [18], Ren et al. [20] and Kim et al. [19] where the authors suggested incomplete reproductive isolation between the two sand lance species. Our study revealed that characteristics of local oceanic current may cause different pattern of gene flows even within the conspecific.

## Supporting information

S1 Table Pairwise Fst values between locations_lineages (below diagonal) and pairwise *P* values (above diagonal) in seven microsatellite loci. (XLSX)

S1 Fig The phylogenetic tree based on mtDNA COI in 444 bp. The Neighbor-joining tree was constructed under the K2P model, and the numbers on branches indicate bootstrap probabilities from 1,000 bootstrap replications.
(TIF)

S2 Fig Allele frequency distributions for five locations of sand lances based on seven microsatellite loci. Each population is indicated by abbreviation; Baengnyeongdo (BA), Tongyeong (TO), Gijang (GI), Jumunjin (JU) and Japan (JP), respectively.
(TIF)

S3 Fig The UPGMA tree based on genetic distance in microsatellite loci. Each population is indicated by abbreviation; Baengnyeongdo (BA), Tongyeong (TO), Gijang (GI), Jumunjin (JU) and Japan (JP), respectively.
(TIF)
