# Supplementary figures and images for: New insight into hybridization and unidirectional introgression between Ammodytes japonicus and Ammodytes heian (Trachiniformes, Ammodytidae)
Source: PLoS One. 2017 Jun 5;12(6):e0178001. doi: 10.1371/journal.pone.0178001 (PMC5459329; doi:10.1371/journal.pone.0178001)

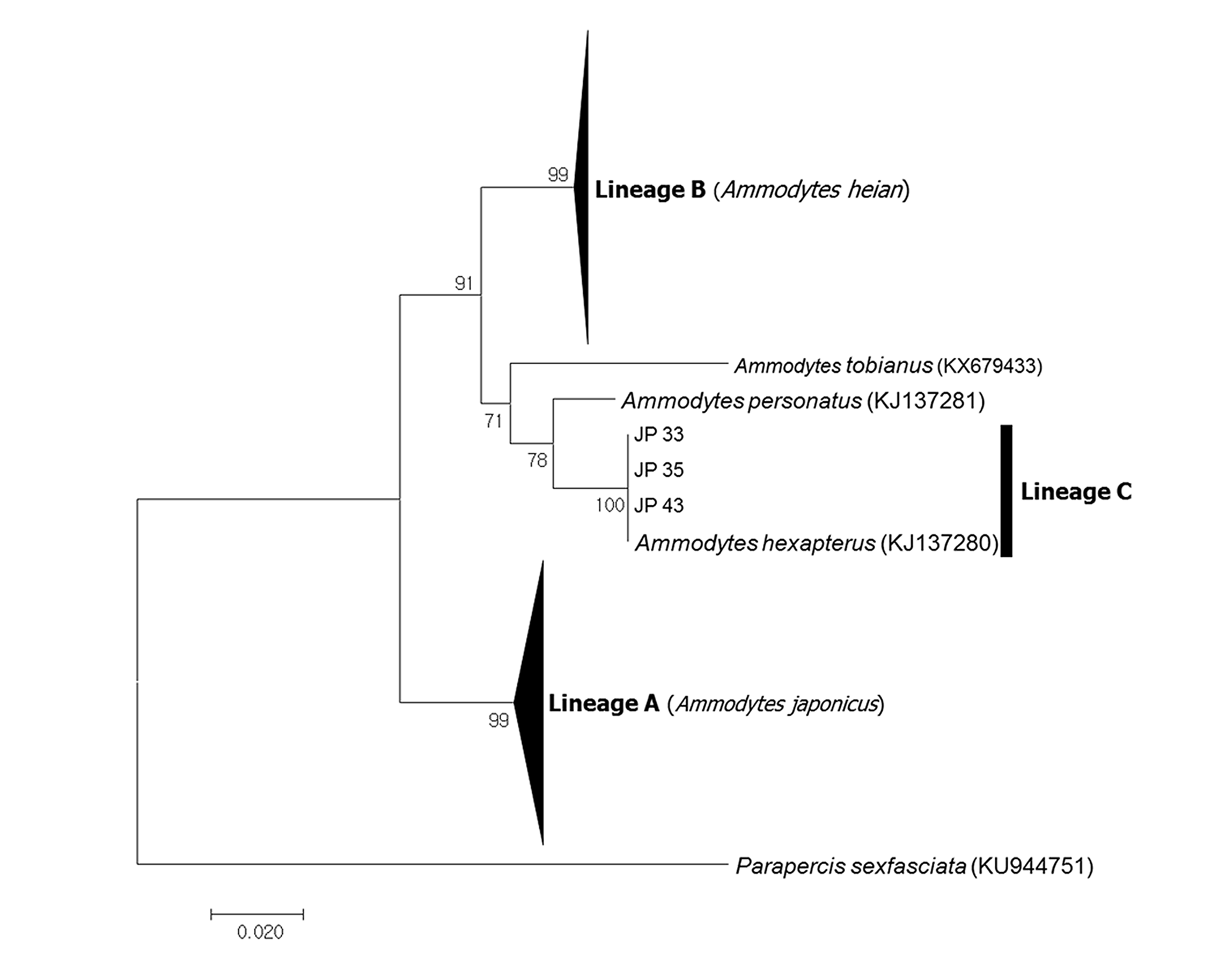

Supplement: S1 Fig — The Neighbor-joining tree was constructed under the K2P model, and the numbers on branches indicate bootstrap probabilities from 1,000 bootstrap replications. (TIF) [file pone.0178001.s002.tif]

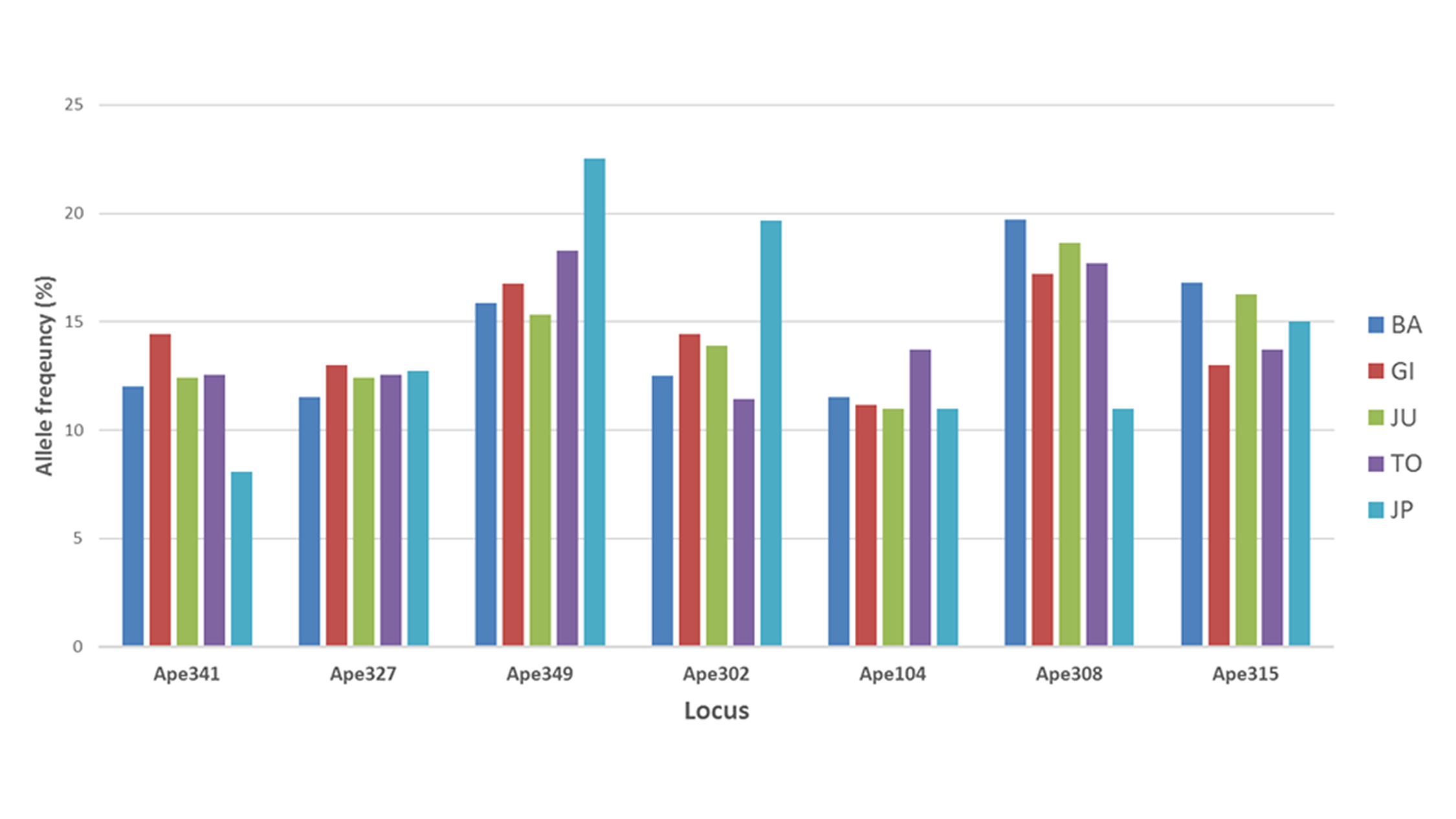

Supplement: S2 Fig — Each population is indicated by abbreviation; Baengnyeongdo (BA), Tongyeong (TO), Gijang (GI), Jumunjin (JU) and Japan (JP), respectively. (TIF) [file pone.0178001.s003.tif]

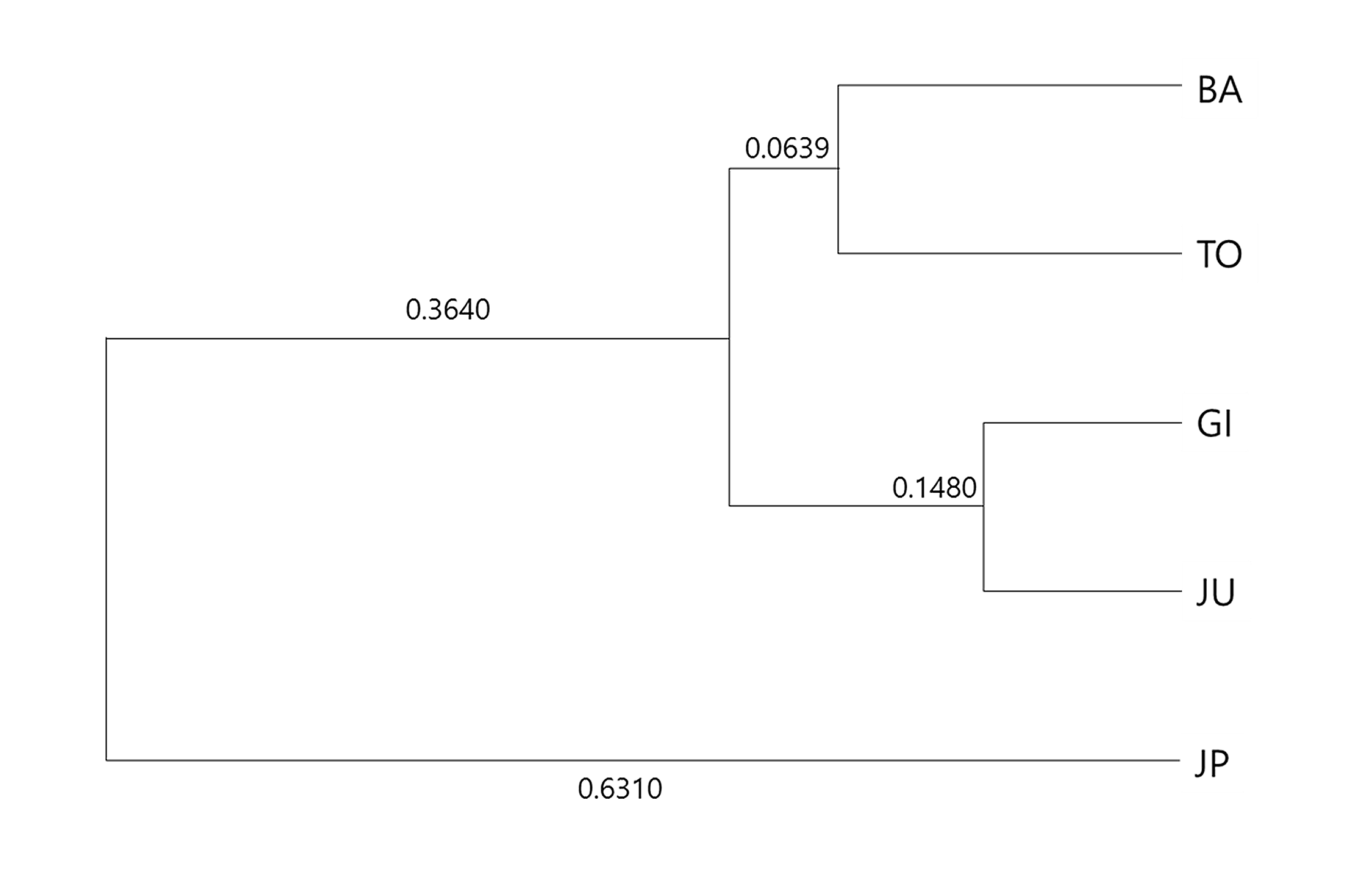

Supplement: S3 Fig — Each population is indicated by abbreviation; Baengnyeongdo (BA), Tongyeong (TO), Gijang (GI), Jumunjin (JU) and Japan (JP), respectively. (TIF) [file pone.0178001.s004.tif]
